# Dual-Bone Grafting Technique of Neck Reconstruction in Non-union Neck Femur Fracture With Avascular Necrosis of the Femur Head

**DOI:** 10.7759/cureus.100236

**Published:** 2025-12-28

**Authors:** Somok Banerjee, Alok Rai, Nirmal Rathi, Suhas Mahendrakar, Rishabh Verma

**Affiliations:** 1 Orthopaedics, All India Institute of Medical Sciences, Raipur, Raipur, IND; 2 Orthopaedics and Trauma, All India Institute of Medical Sciences, Raipur, Raipur, IND

**Keywords:** avascular necrosis, cannulated cancellous screw fixation, femoral neck non-union, fibular strut graft, hip-preserving surgery, sickle cell disease, tricortical iliac crest graft

## Abstract

A 36-year-old male farmer with known sickle cell disease presented four months after a road traffic accident with right hip pain and difficulty walking. He had initially received conservative treatment with traction but discontinued it due to financial constraints. Clinical examination revealed signs of hip instability, a 3 cm true limb shortening, and a Harris Hip Score of 38. Imaging showed a Sandhu Stage 3 non-union of the femoral neck with >50% neck resorption and Ficat-Arlet Grade 2a avascular necrosis (AVN). Given the relatively favorable prognostic indicators, including a Kerboul angle of 100°, the patient opted for a joint-preserving procedure to retain his ability to squat and perform manual labor.

The patient underwent osteosynthesis through a modified Heuter approach. A 3 cm tricortical iliac crest graft was used to bridge the fracture gap and restore neck length, supported by a fully threaded cannulated cancellous (CC) screw. A 7 cm non-vascularized fibular strut graft was introduced across the fracture and further stabilized using a Pauwels screw and an additional CC screw. Postoperatively, he was mobilized non-weight-bearing for six weeks, followed by gradual weight-bearing. Radiographs at six months showed union, and at three years, the patient had minimal shortening, no pain, resumed full activities, and achieved a Harris Hip Score of 85.

## Introduction

Non-union of the femoral neck fractures with avascular necrosis (AVN) of the femoral head represents one of the most difficult scenarios in orthopaedic trauma surgery, particularly in young and active individuals [1]. These cases are further complicated in patients with underlying hematological disorders such as sickle cell disease, which independently contributes to the compromised vascularity of the femoral head. Management decisions in such situations require a nuanced approach to balance hip preservation and functional recovery. While total hip arthroplasty (THA) offers predictable results in older patients, it poses long-term challenges in young individuals due to increased mechanical demands and higher revision rates. Osteosynthesis with bone grafting remains a viable option in select young patients, aiming to preserve native anatomy and optimize biomechanics. We present a case of a young male manual laborer with sickle cell disease who sustained a femoral neck fracture, progressed to non-union with Ficat-Arlet Grade 2a AVN, and was successfully treated with open reduction and internal fixation (ORIF) combined with a dual-bone grafting technique, comprising autologous tricortical iliac crest graft and non-vascularized fibular strut graft, stabilized using cannulated cancellous screws (CC screws) and a Pauwels screw.

## Case presentation

A 36-year-old male farmer presented four months after a road traffic accident, initially managed with skin traction at a district hospital. He had a known history of sickle cell disease and complained of intermittent right hip pain even before the trauma. Due to financial limitations, he delayed further intervention and presented with progressive pain, limp, and inability to bear weight.

On examination, he exhibited a 3-cm limb shortening, external rotation deformity of the lower limb, positive Trendelenburg and telescopy tests, and inability to perform a straight leg raise. No neurological deficits were noted. The Harris Hip Score at presentation was 38.

**Figure 1:**
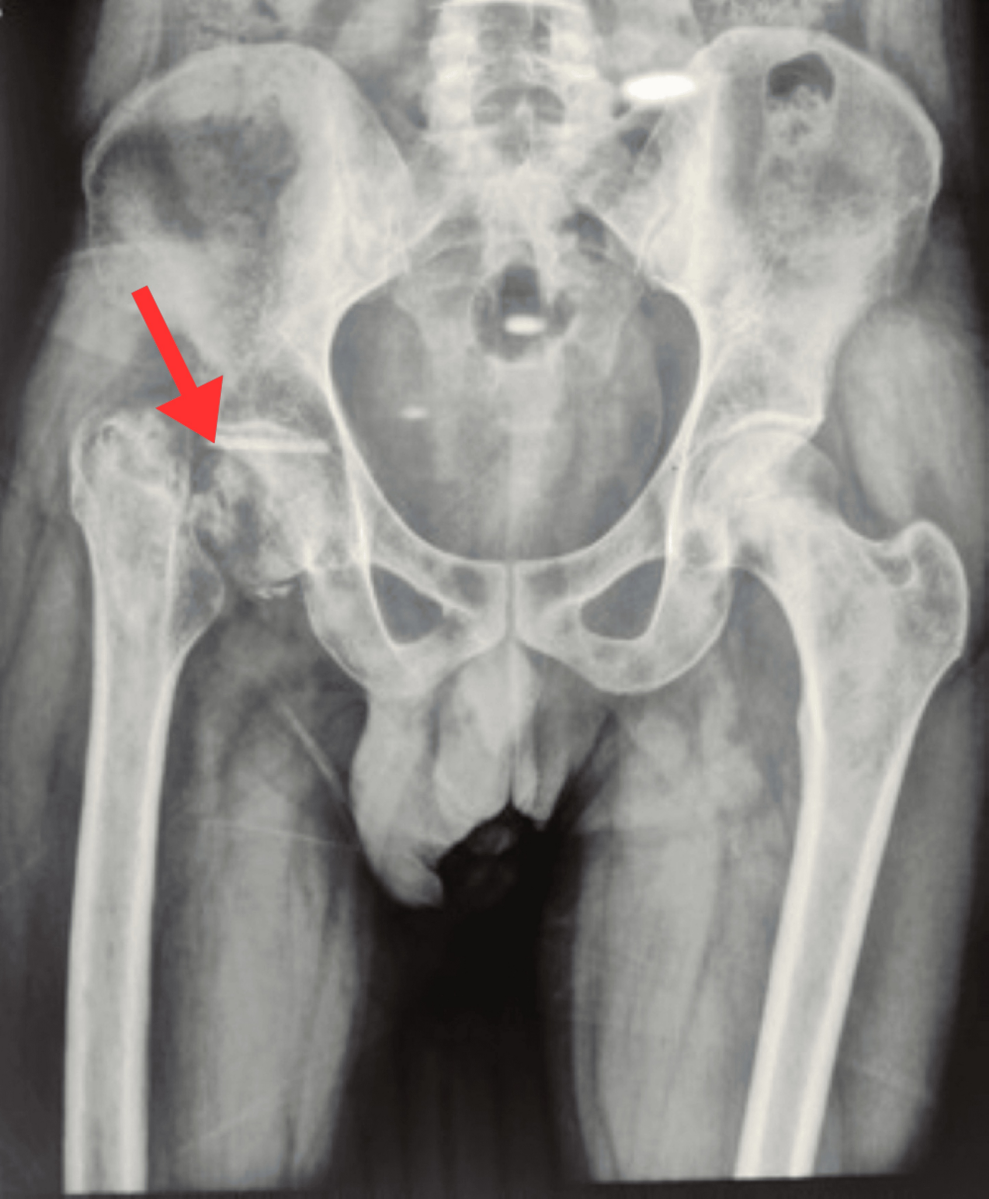
Preoperative radiograph The red arrow indicates non-union of the femoral neck with proximal migration of the greater trochanter.

**Figure 2:**
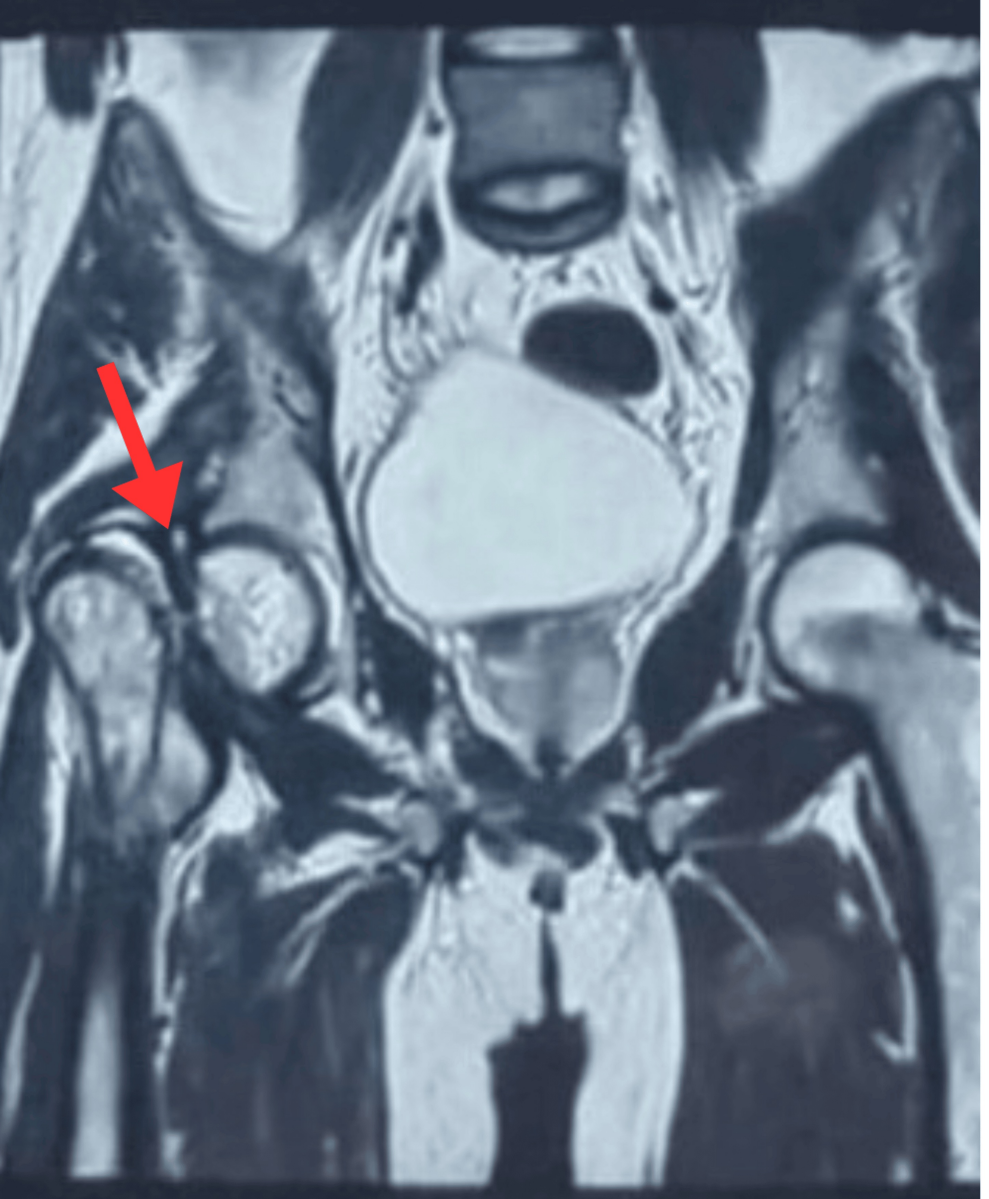
Sagittal section of MRI showing non-union of neck of femur fracture The red arrow indicates non-union of the femoral neck and partial resorption of neck.

Radiographs demonstrated a non-union of the femoral neck with proximal migration of the greater trochanter and partial neck resorption (Figure 1). Magnetic resonance imaging revealed Grade 2a AVN changes in the antero-superior-medial femoral head and a Sandhu Stage 3 non-union (Figure 2). Given the patient’s occupation, functional demands, and desire to avoid the restrictions associated with total hip arthroplasty, he elected for a joint-preserving approach with ORIF and autologous bone grafting.

Surgical technique

After obtaining informed consent, the patient underwent surgery under combined spinal-epidural anaesthesia with prophylactic antibiotics. The patient was positioned supine on a fracture table with traction applied to the right lower limb and a modified Heuter approach was used. A 10-cm incision was made starting 2 cm distal and lateral to the anterior superior iliac spine and extended toward the lateral femoral condyle. The tensor fascia lata was dissected carefully to avoid damaging the lateral femoral cutaneous nerve. The ascending branches of the lateral circumflex femoral artery were ligated. The anterior hip capsule was exposed, and a T-shaped capsulotomy was performed.

On inspection, a 3-cm gap was found at the fracture site, with sclerosed margins. The fracture ends were curetted and freshened. A 3-cm wedge-shaped tricortical iliac crest graft was harvested and used to bridge the defect and restore neck length. The reduction was temporarily stabilized with three K-wires. A 6.5-mm fully threaded CC screw was inserted inferiorly through a separate lateral incision. A slot was created superior to this screw using a triple reamer, and a 7-cm non-vascularized fibular strut graft, harvested from the ipsilateral leg, was placed into the neck bridging the fracture site.

**Figure 3:**
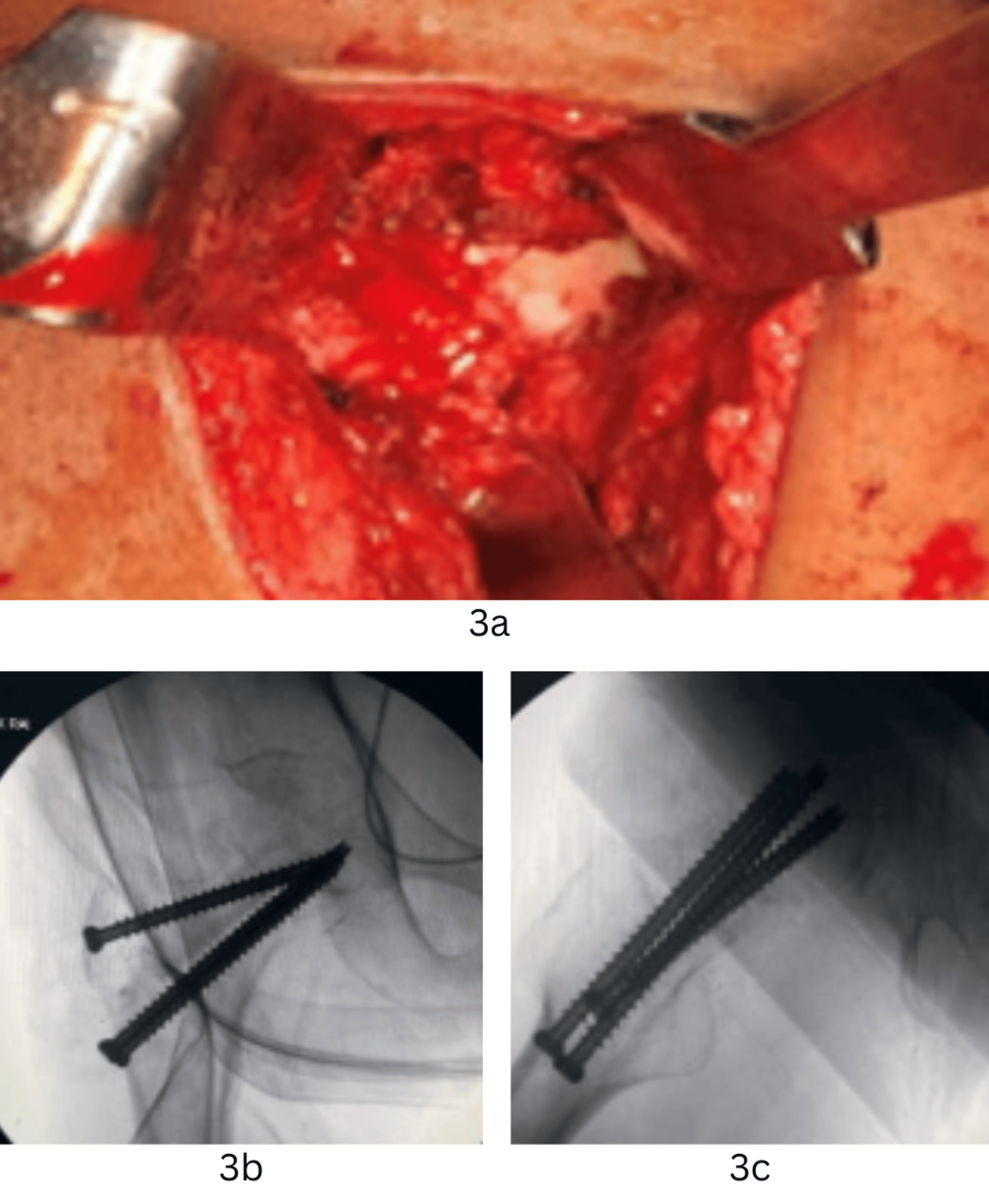
Intraoperative images (3a) Intraoperative clinical photo; (3b) antero-posterior image of final fixation; (3c) lateral image of final fixation.

Additional fixation was achieved with a Pauwels screw and a posteriorly placed 6.5-mm CC screw (Figure 3). Haemostasis was secured, the capsule closed, and the wound closed in layers over a drain. Immediate postoperative X-rays confirmed anatomical reduction and stable fixation.

Postoperative management and outcome

Postoperative rehabilitation included early passive mobilization and isometric exercises. Toe-touch weight-bearing began at six weeks, progressing to full weight-bearing at 6 months after radiographic evidence of union.

**Figure 4:**
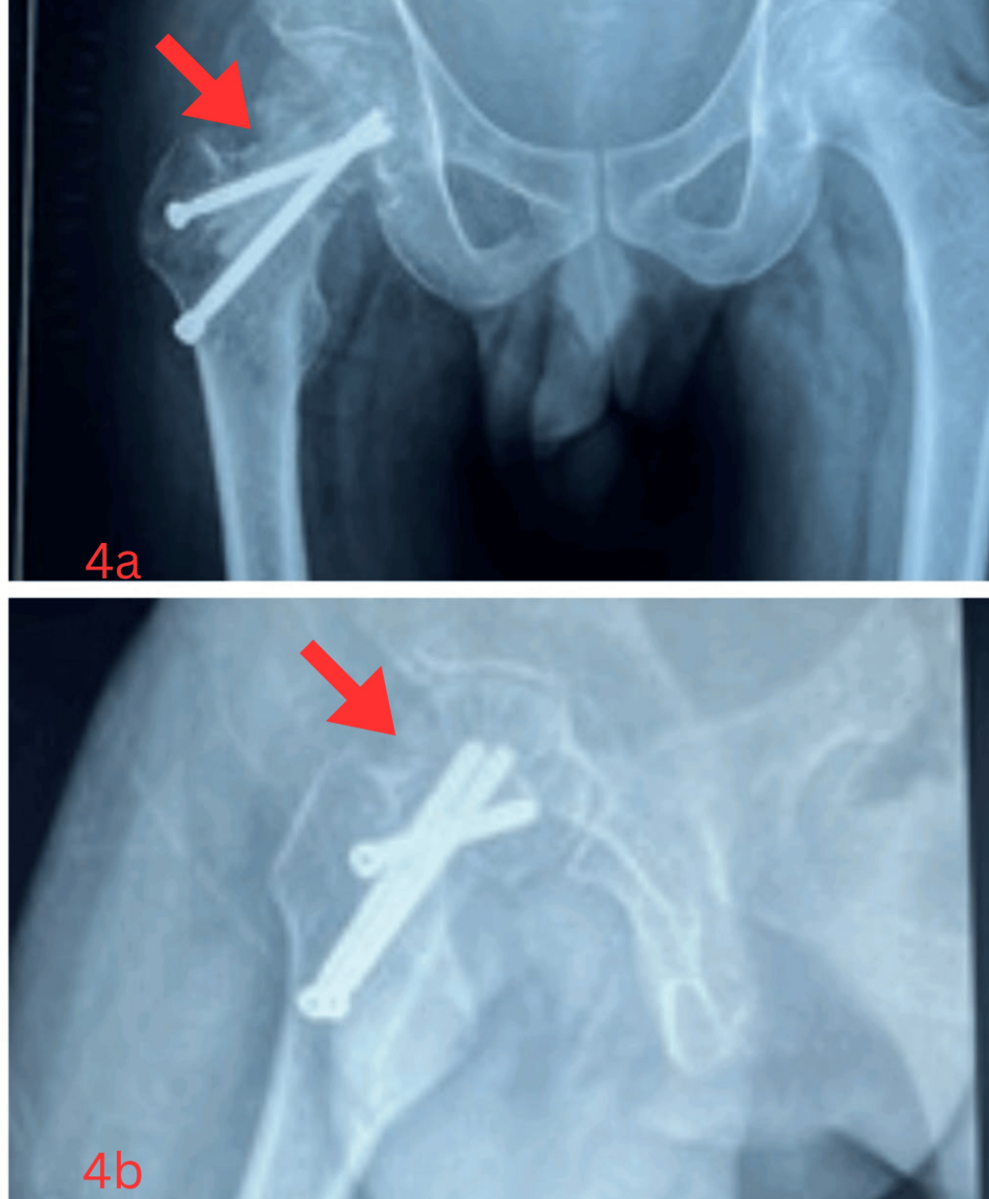
Follow-up orthogonal radiographs after three years The red arrow indicates union at fracture site after three years in antero-posterior (4a) and lateral (4b) radiographs. (4a) Antero-posterior view showing union at fracture site and rigid fixation; (4b) lateral view showing union at fracture site and rigid fixation,

Follow-up radiographs (one-year follow-up) showed gradual graft incorporation and union across the fracture site. At three years follow-up, the patient demonstrated full union (Figure 4) with 1 cm residual shortening. He resumed agricultural activities and was able to squat, sit cross-legged, and ambulate without support. Harris Hip Score improved to 85 from 38, indicating significant functional recovery.

## Discussion

Femoral neck fractures in young adults are often termed “unsolved fractures” due to their tendency toward non-union and AVN. The femoral head’s limited blood supply, particularly from the retinacular vessels, predisposes it to necrosis following fracture. Factors like displacement, vertical fracture orientation (high Pauwels angle), posterior comminution, poor reduction, and delayed treatment contribute to adverse outcomes [2].

**Figure 5:**
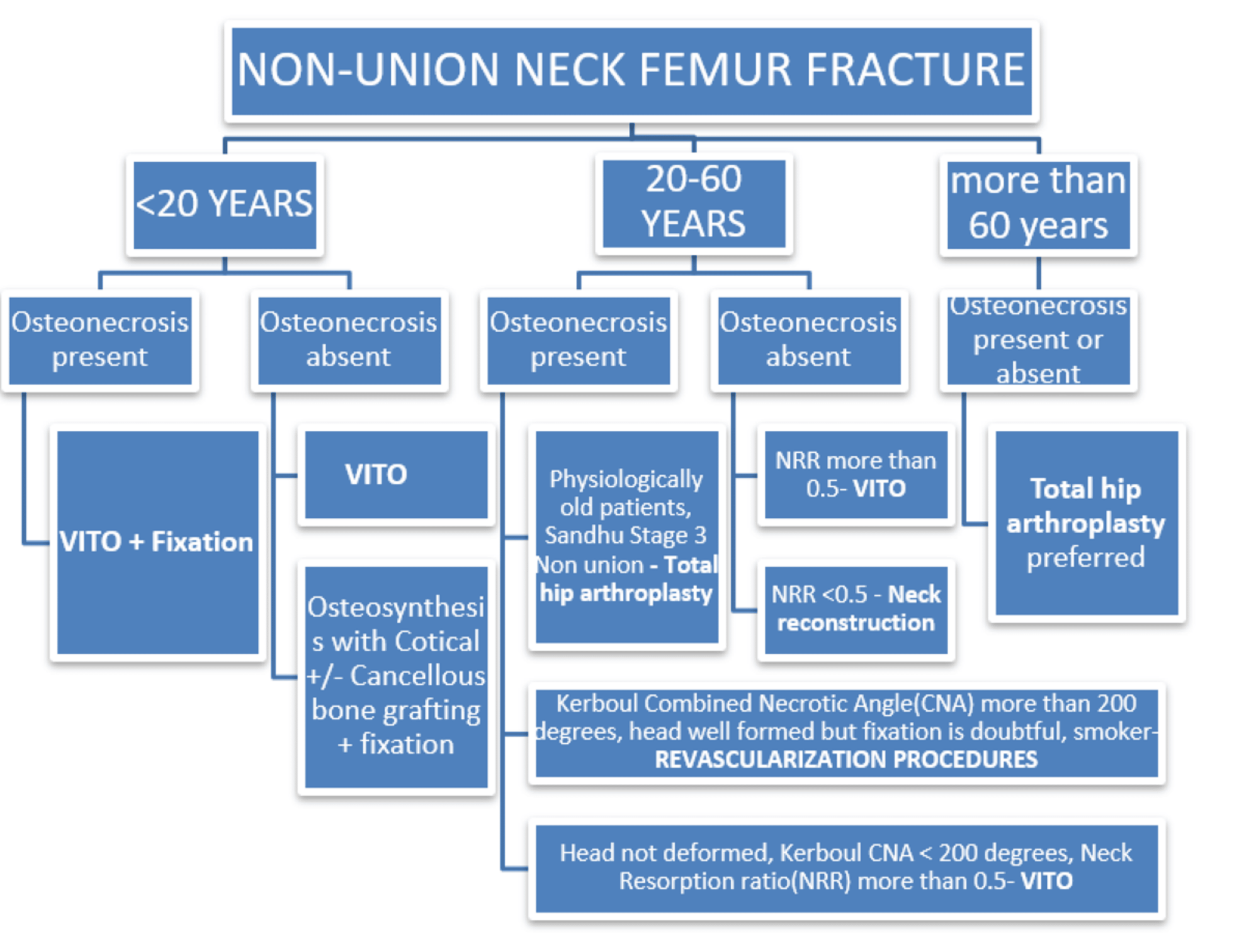
Decision-making algorithm in the management of non-union neck femur fractures Revascularization procedures: Vascularized fibula grafting Kerboul combined necrotic angle: Sum of arc angles subtended by necrotic foci in head of the femur in antero-posterior and lateral views of MRI. Neck reconstruction: All India Institute of Medical Sciences (AIIMS) box technique, Modified AIIMS box technique, etc. Salvage options: Hip arthrodesis in young (not favored), total hip arthroplasty. VITO: Valgus intertrochanteric osteotomy; NRR: Size of femur head remnant/size of femur neck on the sound side (NRR<0.5 indicates high chance of failure from VITO). Credit: Algorithm created by Dr. Somok Banerjee.

AVN further complicates management, especially in sickle cell disease, which impairs microvascular perfusion. Non-union with AVN can lead to mechanical failure, persistent pain, and disability [3]. Treatment choices must weigh preserving native joint anatomy versus the reliability of joint replacement (Figure 5) [4-6]. While THA and hemiarthroplasty offer predictable outcomes in elderly patients, young, active individuals benefit from preserving their biological hip structure [7].

Several surgical strategies exist for non-union femoral neck fractures with AVN, including valgus osteotomy, vascularized or non-vascularized bone grafting, and arthroplasty. Each carries specific indications and complications. ORIF with bone grafting, particularly when combined with strut grafts, offers biomechanical and biological advantages. The iliac crest graft provides osteogenic and osteoinductive potential, while the fibular strut acts as a mechanical buttress, resisting shear forces and facilitating revascularization of the femoral head [4,8].

In our case, the Kerboul angle was <100°, suggesting limited femoral head necrosis, which is associated with better outcomes. Studies suggest that necrotic angles <200° and neck resorption ratios >0.5 correlate with successful osteosynthesis. The dual grafting technique restored length and provided biological and mechanical support, contributing to union and functional success [9].

Literature indicates that ORIF in similar settings can achieve union rates of 60%-80% if anatomic reduction and stable fixation are obtained. Compared to THA, which carries long-term risks of wear, dislocation, and revision surgeries, ORIF enables young patients to maintain native biomechanics and return to high-demand activities [10].

## Conclusions

Femoral neck non-union with AVN in young adults poses a significant challenge. This case demonstrates that ORIF with dual autologous bone grafting using tricortical iliac crest and non-vascularized fibula strut grafts can provide excellent outcomes in carefully selected patients. With proper technique, this hip-preserving method can yield a stable union and restore function in high-demand individuals.
